# Intellectual Structure and Evolutionary Trends of Precision Medicine Research: Coword Analysis

**DOI:** 10.2196/11287

**Published:** 2020-02-04

**Authors:** Xiaoguang Lyu, Jiming Hu, Weiguo Dong, Xin Xu

**Affiliations:** 1 The Department of Gastroenterology Renmin Hospital of Wuhan University Wuhan China; 2 School of Information Management Wuhan University Wuhan China; 3 Center for the Study of Information Resources Wuhan University Wuhan China; 4 The Intensive Care Unit of Coronary Heart Disease Renmin Hospital of Wuhan University Wuhan China

**Keywords:** precision medicine, topics distribution, correlation structure, evolution patterns, coword analysis

## Abstract

**Background:**

Precision medicine (PM) is playing a more and more important role in clinical practice. In recent years, the scale of PM research has been growing rapidly. Many reviews have been published to facilitate a better understanding of the status of PM research. However, there is still a lack of research on the intellectual structure in terms of topics.

**Objective:**

This study aimed to identify the intellectual structure and evolutionary trends of PM research through the application of various social network analysis and visualization methods.

**Methods:**

The bibliographies of papers published between 2009 and 2018 were extracted from the Web of Science database. Based on the statistics of keywords in the papers, a coword network was generated and used to calculate network indicators of both the entire network and local networks. Communities were then detected to identify subdirections of PM research. Topological maps of networks, including networks between communities and within each community, were drawn to reveal the correlation structure. An evolutionary graph and a strategic graph were finally produced to reveal research venation and trends in discipline communities.

**Results:**

The results showed that PM research involves extensive themes and, overall, is not balanced. A minority of themes with a high frequency and network indicators, such as Biomarkers, Genomics, Cancer, Therapy, Genetics, Drug, Target Therapy, Pharmacogenomics, Pharmacogenetics, and Molecular, can be considered the core areas of PM research. However, there were five balanced theme directions with distinguished status and tendencies: Cancer, Biomarkers, Genomics, Drug, and Therapy. These were shown to be the main branches that were both focused and well developed. Therapy, though, was shown to be isolated and undeveloped.

**Conclusions:**

The hotspots, structures, evolutions, and development trends of PM research in the past ten years were revealed using social network analysis and visualization. In general, PM research is unbalanced, but its subdirections are balanced. The clear evolutionary and developmental trend indicates that PM research has matured in recent years. The implications of this study involving PM research will provide reasonable and effective support for researchers, funders, policymakers, and clinicians.

## Introduction

### Background

Precision medicine (PM), also called personalized medicine, is a new medical model aimed at providing precise diagnosis, therapy, prognosis prediction, and prevention strategies based on information in a patient’s genes, proteins, and their environment [1]. The scientific basis of PM is molecular pathological epidemiology, and it aims to identify the relationship between biomarkers, the drug response, and outcome in disease [2,3]. During the Human Genome Project, it took “one dollar one bp” and 13 years to complete the sequencing of the whole genome. Owing to breakthroughs in techniques and lower prices, effective, high-throughput, and accurate sequencing can be applied to map genomics, metabolomics, microbiomics, and proteomics, which has led to the discovery of increasingly more causative biomarkers [4-7]. However, clinicians currently use clinical trials and pilot studies to assess the relationship between biomarkers and diseases.

Great progress has been made in personalized treatment in the field of oncology. According to a meta-analysis of phase II clinical trials, a personalized treatment strategy across malignancies yields a better outcome and lower likelihood of death than nonpersonalized targeted therapies [8]. Thus, it can be expected that PM will use new knowledge, including the integration of clinical medicine, pathology, epidemiology, and omics, to provide better therapies for patients.

Owing to the potential importance of PM, a few leading experts reviewed this new medical approach in regards to its relevant history, clinical applications, and any interdisciplinary research associated with PM, such as bioinformatics, artificial intelligence, and big data [9-11]. It is logical to assess the status of the subfields or branches. However, there are still some limitations regarding the review themes; specifically, the overall structure and characteristics of PM research have not been mapped, the relationship between the subfields has not been revealed, and the predictions regarding PM in those reviews were not made based on an accurate quantitative analysis.

Coword analysis is a bibliometric method used to identify relationships between subfields within research areas and to measure the strength of the relationships [12,13]. According to the co-occurrence correlation, the keywords can be classified into clusters and displayed as network maps. Some other indices, such as density and centrality, can be used to evaluate the shape of the maps. By comparing the network maps of different periods, the dynamic evolution of one research area can be clearly displayed. Owing to the characteristics of “quantitative” and “zoom” in coword analysis, scientists can uncover the links within a subfield, obtain the overall structure of networks according to simplified graphs, and focus on one certain subarea to obtain more information [13].

Coword analysis has been widely used to illustrate the intellectual structure and developmental status of research areas [14-16]. Our study applied this method to explore the overall research structure, correlation among themes, and entire set of evolutionary trends in the field of PM. Our results may help scientists and clinicians better understand its developmental characteristics and even yield new insights on breakthroughs.

### Literature Review

PM is a new medical approach that classifies patients into different groups related to their diagnosis, treatment, and prevention based on individual gene, protein, or environmental information. It is noteworthy that the terms “precision medicine,” “personalized medicine,” “stratified medicine,” and “P4 [predictive, preventative, personalized, and participatory] medicine” are still interchangeably used by some organizations and scientists [17,18]. In the period after the Human Genome Project, considerably more effort was put into exploring the relationship between genomic information and patient care [19]. In 2015, the Precision Medicine Initiative was launched by Barack Obama, then the president of the United States, indicating the beginning of a new medical age [20].

Every person has polymorphisms in their DNA, RNA, and proteins, as well as methylation. Recent scientific methods have enabled the analysis of biomarkers using omics techniques, including genomics, epigenomics, transcriptomics, proteomics, metabolomics, microbiome analysis, and immunomics. However, pathologists, epidemiologists, and clinicians have also made many contributions to the discovery of links between biomarkers and clinical features [21]. Advances in the PM model have played an important role in disease diagnosis, treatment, and prevention. Cancer treatment is the field in which PM originated and it has seen the most mature use of PM, such as when cancer genomics were successfully applied in personalized medicine. With the deep awareness of genetic variations of tumors, treatment strategy can be tailored to the group of cancer patients with the same genotype. For example, the human epidermal growth factor receptor 2 (HER2) gene is amplified and overexpressed in 25-30% of breast cancers. According to evidence from clinical trials, trastuzumab, a targeted therapy drug, increases the clinical benefit of first-line chemotherapy in metastatic breast cancer that overexpresses HER2 [22].

PM also plays an important role in disease treatment and prevention. DNA information from individual phenotyping will lead to more effective and accurate treatment and prevention. For example, the high risk in women for developing breast cancer is strongly correlated with mutations in *BRCA1* or *BRCA2* [23]. Clinicians can make better decisions regarding prevention for patients carrying these genetic mutations. Pharmacologists and genomic scientists have also provided many contributions to the assessment of genetic variations that affect drug discovery and clinical pharmacology [24]. Considering information on personal phenotypes, physicians can provide reasonable drug prescriptions that represent targeted therapies and are more cost-effective, but also have fewer side effects [25]. Furthermore, PM has changed clinical trials. Among the minority of clinical trials involving PM, the proportion of trials on adult cancers in the United States that require a genomic alteration for enrollment has increased substantially over the past several years [26]. Finally, PM will yield some challenges in the field of ethics, patient privacy, and refurbishment policies, which will require more attention from scientists and policymakers in the field [27,28].

### Previous Efforts

With the pace of PM research rapidly increasing, a large number of studies have been performed from different perspectives. Many reviews have been published to facilitate a better understanding of the status of PM research, as well as clarifying the concept, history, clinical application, ethical concerns, and technological challenges. The efforts listed above have helped raise awareness of the new clinical model among patients, clinicians, and even health policy makers. They have played an important role in the development of intelligent support for decision-making, clinical practice, and public health policies.

According to recent reviews, the features of PM research are as follows. First, some reviews reported by top experts in the field of PM research discuss the foundation, techniques, applications, and perspectives of this new discipline [4,20,29]. Second, PM research involves significant interdisciplinary collaboration. Many scientists, such as those specializing in clinical medicine, clinical oncology, systems biology, or biochemistry, are focused on the development of this new field. Advanced technologies, such as next-generation sequencing (NGS), molecular imaging, omics (genomics, proteomics, metabolomics, and microbiomics), nanotechnology, big data, and artificial intelligence, have been applied to laboratory tests in PM to achieve more accurate results [6,7,30-34]. Third, the scope of the PM model has been expanded from clinical oncology to noncancer disciplines. This strategy has led to several innovations in the diagnosis and treatment of mental illness, cardiovascular disease, asthma, and inflammatory bowel disease [35-38]. Finally, the reviewers also emphasized the issues to be solved in the development of PM, such as technological bottlenecks, patient privacy, and ethical challenges [39-41]. The information provided in the reviews made a large contribution to the global acknowledgment of PM.

### The Rationale for the Study

Research on PM is still increasing, and some important discoveries have already been beneficial to patients. However, there is still a long way to go in the utilization of PM. How can interdisciplinary researchers start studies? What type of public policy really makes sense regarding the field? How can funders ensure that investment works effectively? All these decisions should be made based on knowledge of PM, so great efforts have been made to describe the nature of this new field. The aim of our study was to address the following problems:

1. What is the distribution of topics in PM research?
2. What is the correlation structure of topics in PM research?
3. What are the evolutionary venations and development trends of PM research?

## Methods

### Data Collection and Processing

**Figure 1:**
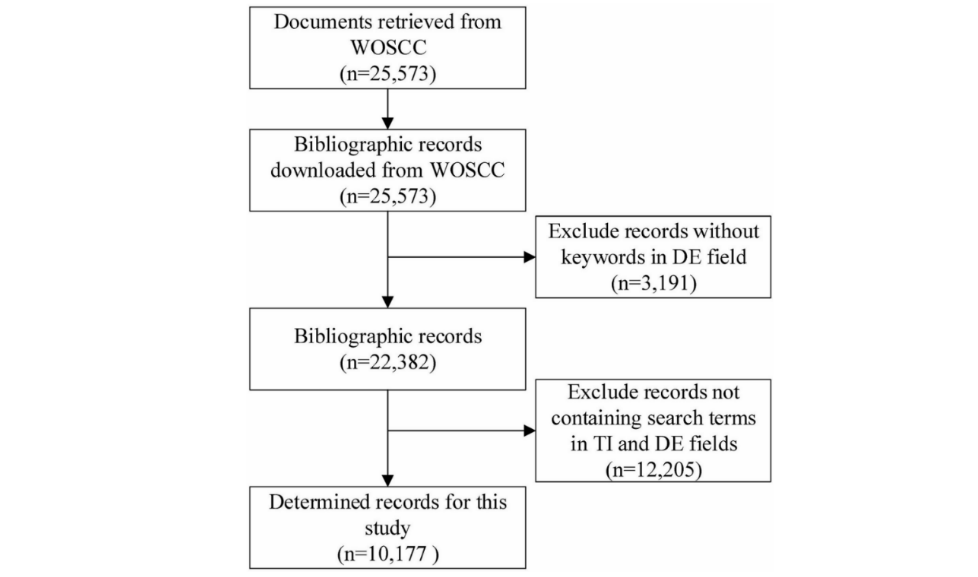
Search procedure for documents in precision medicine research. DE: descriptor; TI: title; WOSCC: Web of Science Core Collection.

According to previous studies, papers in the Web of Science Core Collection (WOSCC) can represent the status of medical science, including PM; therefore, we chose WOSCC as our data source. Data processing is shown in Figure 1.

Papers were collected from the WOSCC that covered the period from 1999 to 2018. In this study, initial retrieval was conducted using “precision medicine,” “P4 medicine,” “personalized medicine,” and “stratified medicine” as terms in the field of Topic to guarantee a recall ratio. It included the document types of Article, Review, and Proceedings. The retrieval strategy is illustrated as follows: TOPIC: (“precision medicine”) or TOPIC: (“personalized medicine”) or TOPIC: (“individualized medicine’’) or TOPIC: (“P4 medicine’’) or TOPIC: (“stratified medicine”). Refined by: Document Types: (Article or Review or Proceedings Paper) Timespan: 1999 to 2018. Indices: SCI-EXPANDED, SSCI, A&HCI, CPCI-S, CPCI-SSH, ESCI, CCR-EXPANDED, and IC.

**Figure 2:**
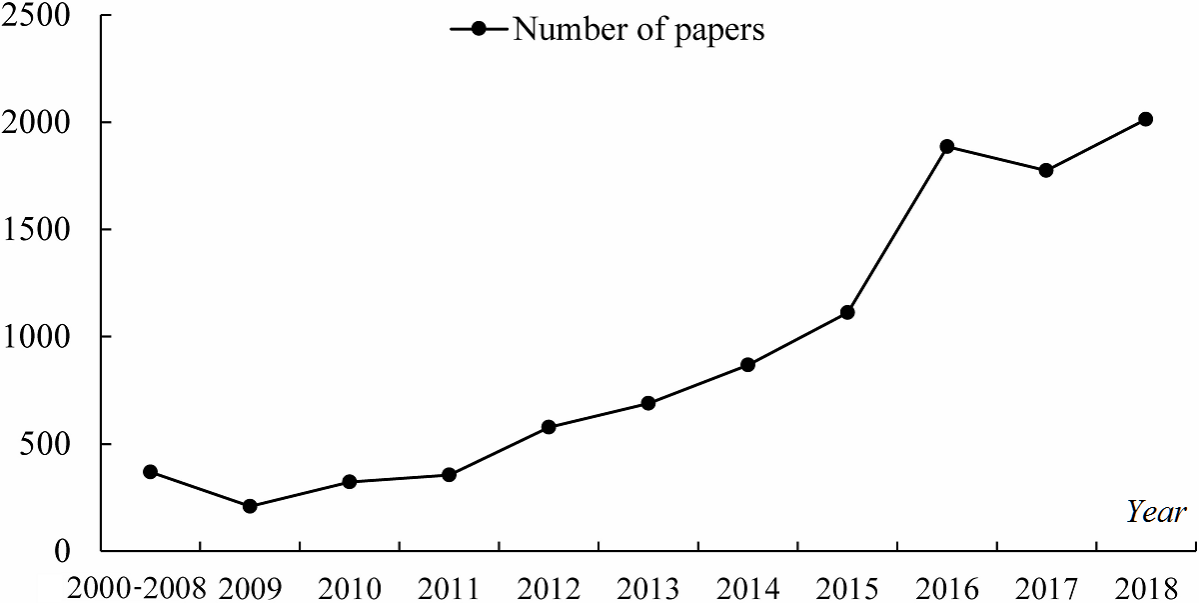
Basic statistics of the sample papers from 2000 to 2018.

A total of 25,573 publications were retrieved, and their bibliographic records were downloaded through the function Save to Other File Formats provided by WOS. Next, a text file containing all records in Tab-delimited (Win) format was obtained. In general, records without keywords data were excluded. Meanwhile, publications not containing the search terms above in Title (the TI field) or Keywords (the DE field) were identified as unrelated to PM research [31] and also excluded. Thus, 10,177 records were selected as the final data sample, and there were 17,818 unique keywords. Figure 2 shows the number of papers in the sample by year. The PM research started proliferating in 2000, and the number of related papers increased each year.

In this study, mainstream keywords of high frequency were selected for further analysis, that is, based on their coword network. The largest connected component extracted from the whole coword network represents the mainstream research directions of one field [42]. After several rounds of testing of the largest connected component using social network analysis, keywords with a frequency of ≥20 were selected. The sum of the frequency of these words accounts for 52.19% (24,492/46,921) of the total that can represent mainstream research of PM. Meanwhile, keywords were normalized to ensure consistent treatment of the singular and plural forms of words, unifying the synonyms and clarifying the homonyms. For example, the items “target, targets, targeting, target-Specific” were replaced by “Targeted Therapy.” Keywords with a frequency of less than 20 were merged into broader terms. For example, “Hematopoietic Stem Cell” (frequency 6) is replaced by “Stem Cell” (frequency 107). General items which were too broad to be of practical connotation, such as “medicine,” “research,” and “mechanism,” were removed. With the replacement, 244 related words with a frequency greater than 20, which were collected as the basic sample for analysis, were used in this study.

### Methodology and Tools

Keywords in a paper provide an adequate description of its contents. A study on the correlation of keywords can reveal connotations of contents [43]. Co-occurrence [13] of words (coword) is a kind of correlation in connotation, that is to say, two keywords co-occurring within the same document (eg, paper) are an implication of correlation between topics which they refer to [44]. The high frequency of co-occurrence of each keyword pair means a high degree of correlation between them. Coword analysis has been proven to be effective in identifying main themes and revealing the intellectual structure and patterns of a research field in many previous studies [42,43]. In this study, we used coword analysis that combines both social network analysis tools and scientific mapping tools to analyze the research field of PM. These tools were used to detect and visualize its overall research structure and patterns, conceptual subdomains (thematic communities), and thematic evolution.

#### Social Network Analysis

Many methods have been used to conduct coword analysis, including the method of social network analysis. It is derived from mathematical graph theory, which computes indicators of a coword network and identifies characteristics of the whole network and an individual network [45]. SCI2, version 1.2 beta (Cyberinfrastructure for Network Science Center, Indiana University, Bloomington, Indiana, United States), is an effective bibliometric tool to extract items (eg, keywords, authors, and institutions) from bibliography records of articles or other structured research literature [46]. Its feasibility and effectiveness has been widely proven in previous studies [31]. In this study, the bibliographic record file was imported into SCI2 to obtain statistical data of keywords and coword network data. Coword network data include both keywords and their links with the respective weights. The weight of a keyword is its frequency of occurrence and that of the link between the keyword pair is its frequency of co-occurrence.

As unconnected or uncorrelated keywords cannot reflect main thematic subdomains and as what we focused on is the largest component [47], we used SCI2 to exclude isolated nodes and extract the largest component of the coword network [48,49]. Network indicators of the largest component of the coword network were then calculated using Pajek [50], including centralization (centrality), density, and the clustering coefficient. Network indicators of the whole network or nodes can be used to identify the overall intellectual structure and patterns of one research field as well as a keyword’s characteristics, such as power, stratification, ranking, and inequality, in the network [31].

Centralization measures the overall characteristics of global network, degree centralization measures the centripetal degree, and closeness centralization measures the proximity degree between any 2 nodes in the network. Its high level equals the close distance between any 2 nodes on the whole. Betweenness centralization indicates the degree of correlation between any 2 nodes through a third one (bridge), and its high level equal the high possibility of correlation through a bridge. Similarly, centrality, the individual network indicator, measures the capacity of one node in network. High degree centrality of one node indicates that it is central in the network and is correlated to many other nodes. It also indicates its powerful capacity of influence and control. High closeness centrality equals the capacity of one node that correlates others as short as possible or directly correlates others. High betweenness centrality equals the powerful role as a bridge to correlate other 2 nodes. Density measures the correlation strength within the network [51]. It means the higher the density, the more mature the research field. The clustering coefficient indicates the possibility that keywords are clustered into a contrasting group [52].

In addition, community detection is an effective method to discover research directions or subfields according to the correlation structure of the network [53]. The Louvain algorithm embedded in Pajek, the most common algorithm used to detect communities, was also used to detect communities in this study [54]. Different communities, including highly correlated keywords, represent different research directions or subfields.

#### Visualization and Evolution Analysis

Visualization is an important method to intuitively display the intellectual structure of coword correlation, the thematic evolution of a research field, and even the comparative development trends of subfields [55,56]. After repeated comparison of several visualization tools, VOSviewer, version 1.6.13, Centre for Science and Technology Studies, Leiden University, Leiden, Netherlands), was found to enable better visualization of topological networks and was selected to conduct the visualization in this study, including the overall network with communities and the individual networks [57]. The research themes of PM have been evolving over time. We divided bibliographic records chronologically and imported them into Cortext [58]. Evolutionary trends of the keyword community were visualized, allowing for a layout of the dynamics as depicted by tubes in an alluvial model [59].

A strategic diagram indicates the comparative status and evolutionary trends of subfields of one research field. It is a two-dimensional (2D) map in which the x-axis represents centrality and the y-axis represents density [60]. The origin of the axes is determined by the average centrality and density. Centrality can be understood as a measurement of importance and the degree of core in the whole research network. Density can be understood as a measurement of maturity of a theme’s development. A total of 4 quadrants in a strategic diagram represent different meanings. Themes in Quadrant 1 are central and developed, with both high centrality and high density; in Quadrant 2, they are highly developed but isolated, with high density and low centrality; in Quadrant 3, they are marginal and isolated (emerging or declining), with both low centrality and low density; and in Quadrant 4, they are central with a trend toward high centrality but low density. Therefore, the developing status and trends of themes or research communities can be predicted by a strategic diagram.

## Results

### Themes Involved in Precision Medicine Research

**Figure 3:**
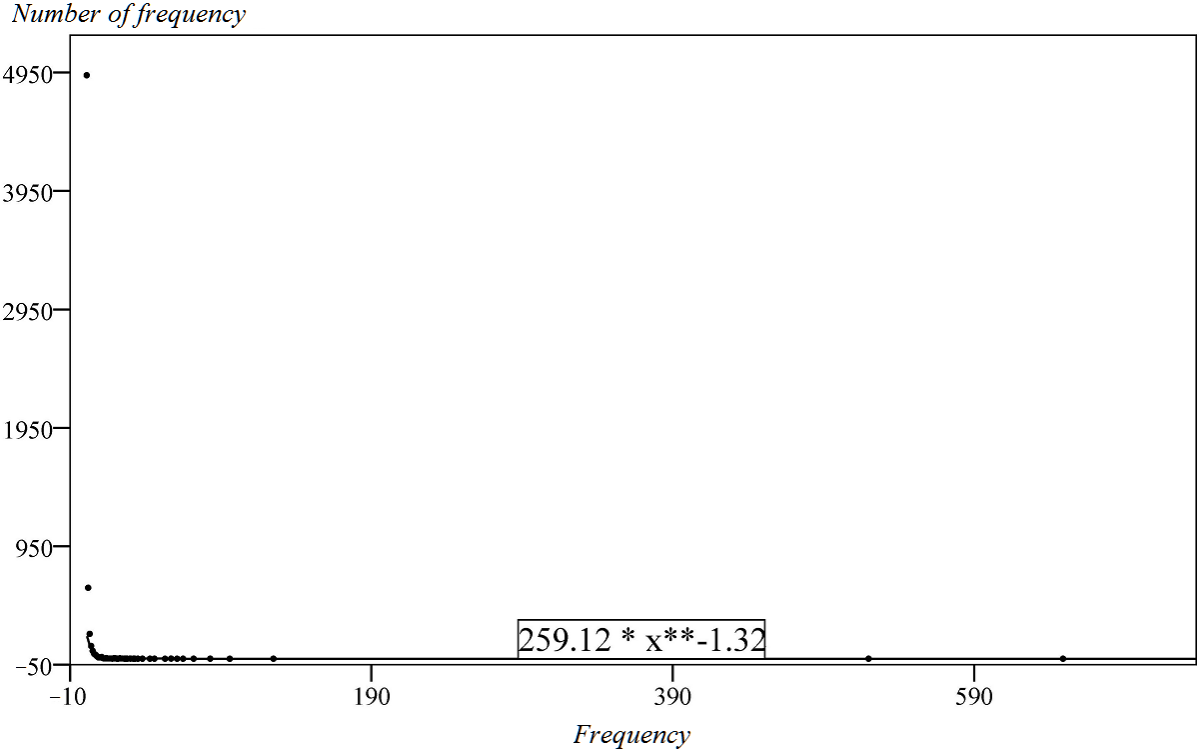
The distribution of the keyword frequency in PM research.

In this study, a total of 17,818 keywords were extracted from the sample, and the total frequency was 47,883. The frequency distribution conforms to the power law distribution with an exponent of –1.32 (Figure 3). This shows that the frequency of very few keywords is very high, whereas most keywords are of extremely low frequency. The results indicate that the subject trends in current PM research are obvious, and researchers are inclined to focus on a few major themes and pay less attention to most other themes in the PM field.

Table 1 lists the 100 most frequent keywords, the sum of the frequencies of which accounts for up to 39% of the total frequency. The keywords are so typical and representative in research topics that they can be considered as the mainstream themes of PM research in the past decade. It is interesting that the proportion of the 10 most frequent keywords is 14.2%. Biomarkers and Genomics are the first echelon; Cancer, Therapy, and Genetics are the second echelon; Drug, Target Therapy, Pharmacogenomics, Pharmacogenetics, and Molecular belong to the third echelon. The findings highlight the core and mainstream of PM research topics and show the imbalanced status of PM research as well.

**Table 1:** Top 100 keywords in precision medicine research.

Number	Keywords	Frequency
1	Biomarkers	1018
2	Genomics	970
3	Cancer	851
4	Therapy	731
5	Genetics	684
6	Drug	549
7	Target Therapy	510
8	Pharmacogenomics	508
9	Pharmacogenetics	475
10	Molecular	357
11	Breast Cancer	333
12	NGS^a^	314
13	Tumor	296
14	Prediction	287
15	Mutation	281
16	Clinical Trials	268
17	Gene	259
18	Sequencing	242
19	Imaging	239
20	Diagnostics	223
21	Proteomics	211
22	Prognosis	209
23	DNA	195
24	Phenotype	187
25	Oncology	185
26	SNP^b^	173
27	Omics	170
28	Pharmacology	165
29	Metabolism	160
30	Lung Cancer	160
31	Bioinformatics	151
32	Asthma	148
33	Chemotherapy	147
34	Immunotherapy	146
35	Stem Cell	143
36	MicroRNA	137
37	Epigenetics	137
38	Prostate Cancer	135
39	Genetic Test	132
40	EGFR^c^	129
41	Risk	129
42	Inflammation	126
43	GWAS^d^	125
44	Polymorphism	124
45	Colon Cancer	123
46	Immune	123
47	Nanotechnology	122
48	PET^e^	122
49	Translation Medicine	120
50	NSCLC^f^	119
51	Heterogeneity	118
52	Big Data	118
53	Systems Biology	117
54	Machine Learning	117
55	Protein	115
56	Pathology	115
57	Genotype	114
58	Ethics	111
59	Health Care	110
60	Drug Development	110
61	Pharmacokinetics	109
62	Drug Delivery	108
63	RNA	105
64	Diagnosis	105
65	Prevention	103
66	Biobank	103
67	Biology	102
68	Patients	100
69	Diabetes	100
70	Theranostics	100
71	Metabolomics	97
72	Liquid Biopsy	97
73	Screening	97
74	Depression	96
75	Classification	95
76	MRI	93
77	Molecular Imaging	92
78	Brain	91
79	Decision Support	91
80	Electronic Health Records	89
81	Systems Medicine	89
82	Resistance	88
83	Cardiology	87
84	Clinical Medicine	87
85	Circulating Tumor Cell	86
86	Pancreatic Cancer	84
87	Companion Diagnostics	83
88	Nanoparticle	83
89	Toxicity	81
90	Radiology	81
91	Mass Spectrometry	79
92	Drug Resistance	77
93	Clinical Practice	75
94	Microarray	74
95	Cell	73
96	Metastasis	72
97	Molecular Diagnostics	70
98	Education	70
99	Gastric Cancer	70
100	Gene Expression	70

^a^NGS: next-generation sequencing.

^b^SNP: Single Nucleotide Polymorphisms.

^c^EGFR: epidermal growth factor receptor.

^d^GWAS: genome-wide association studies.

^e^PET: positron emission tomography.

^f^NSCLC: non–small cell lung cancer.

### Correlation Network Analysis of Precision Medicine Research

#### Network Indicators of the Correlation Structure of the Themes

The 244 keywords (frequency above 20) in the study generate a total of 9178 edges, which constitute a keyword correlation network. It is known that the network is the largest connected component, indicating that a relatively consistent mainstream direction has been formed in PM studies in recent years. As shown in Table 2, the degree centralization and closeness centralization of the keywords are relatively high, indicating that the overall network is more concentric, and most of the keywords are clustered, centering on a few core keywords. We also discovered that the keywords in the network tend to be directly correlated rather than indirectly correlated. The path between keywords is short and tends to be directly correlated with the core words. Therefore, it can be concluded that a few core words have very strong control of the entire network. According to the characteristics listed above, we can draw the following conclusions: current PM research is very centralized, the difference between the core words and noncore words is obvious, and the main themes are formed around the core words. However, the lower betweenness centrality also indicates that most of the keyword correlations in PM research can form direct correlations without other words working as bridges. Combined with higher clustering coefficients, it can be observed that there are multiple thematic directions in this PM study with a large degree of difference. The correlation between keywords within the subject direction is higher than that between the other directions. Finally, the overall network is closely correlated, equaling a high network density. This result means that PM research has formed a systematic, relatively mature research pattern.

In the same way, the indices used to describe each keyword (degree centrality, closeness centrality, and betweenness centrality) represent their position and role in the network. As shown in Table 3, Table 4, and Table 5, the keywords, such as Biomarkers, Genomics, Therapy, Cancer, Genetics, Drug, Prediction, Pharmacogenomics, Target Therapy, and Molecular, occupied the top 10 positions on the lists of degree centrality and closeness centrality. It is particularly worth mentioning that the orders of the keywords in the lists of degree centrality and closeness centrality are identical, and both indicators are of high value. These words are clustered around a large number of keywords to a large extent, which are themselves directly correlated with other keywords. This indicates that these keywords are very important, are in the core position, and have a strong influence on the entirety of PM research. In contrast, the value of betweenness centrality is low. Instead of using an intermedia or a bridge word, most keywords are directly correlated, and the connection path is short. Interestingly, the ranking of the keyword “Therapy” is significantly improved in the list of betweenness centrality compared with its position in the lists of degree centrality and closeness centrality. It indicates that “Therapy” plays an important role of bridging other keywords in the overall network in PM research.

**Table 2:** The statistics of the correlation network in precision medicine research.

Indicators	Value
Number of nodes	244
Number of edges	9178
Average degree	75.2295
Network all degree centralization	0.6214
Network all closeness centralization	0.6685
Network betweenness centralization	0.0277
Network clustering coefficient	0.4843
Density	0.3096

**Table 3:** Top 10 keywords in terms of degree centrality.

Ranking	Keywords	Degree
1	Biomarkers	225
2	Genomics	222
3	Therapy	220
4	Cancer	215
5	Genetics	213
6	Drug	208
7	Prediction	184
8	Pharmacogenomics	183
9	Target therapy	177
10	Molecular	172

**Table 4:** Top 10 keywords in terms of closeness centrality.

Ranking	Keywords	Closeness
1	Biomarkers	0.9310
2	Genomics	0.9205
3	Therapy	0.9135
4	Cancer	0.8967
5	Genetics	0.8901
6	Drug	0.8741
7	Prediction	0.8046
8	Pharmacogenomics	0.8020
9	Target therapy	0.7864
10	Molecular	0.7739

**Table 5:** Top 10 keywords in terms of betweenness centrality.

Ranking	Keywords	Betweenness
1	Therapy	0.0305
2	Biomarkers	0.0304
3	Genomics	0.0304
4	Drug	0.0289
5	Genetics	0.0283
6	Cancer	0.0260
7	Pharmacogenomics	0.0182
8	Prediction	0.0166
9	Target therapy	0.0148
10	Gene	0.0135

#### The Themes of the Correlated Communities

On the basis of community detection in the coword network, PM research has focused on 5 theme communities or research subdirections in the last decade. These communities are visualized as Figure 3 in the next section. Modularity (0.2077) [61] of community detection indicates a good result to distinguish topic communities in PM research. Each community has a strong internal correlation, and the distinction between them is obvious. These communities are as follows: C1-Cancer (including Target Therapy, Molecular, Breast Cancer, NGS, Tumor, Mutation, Clinical Trials, Gene, and Prognosis), C2-Biomarkers (including Prediction, Diagnostics, Proteomics, Phenotype, Omics, Metabolism, Bioinformatics, Asthma, and Inflammation), C3-Genomics (including Genetics, Sequencing, Epigenetics, Genetic Test, Risk, Genome-Wide Association Studies, Translation Medicine, Ethics, and Health Care), C4-Drug (including Pharmacogenomics, Pharmacogenetics, Single Nucleotide Polymorphisms, Pharmacology, Polymorphism, Genotype, Drug Development, Pharmacokinetics, and Depression), and C5-Therapy (including Therapy, Imaging, Stem Cell, Nanotechnology, positron emission tomography [PET], Drug Delivery, Theranostics, MRI, Molecular Imaging, and Brain). According to the research scale, PM studies can be divided into 3 levels: Level 1, C1, is the largest level; Level 2, including C2, C3, and C4, is the medium scale; and Level 3, C5, is the smallest. On the basis of the results above, the study of PM mainly focused on Cancer, Biomarkers, Genomics, and Drug in the past decade. More importantly, these themes represent the mainstream direction of PM studies; however, the C5-Therapy community is still weaker than the other 4 communities.

#### Visualization of the Theme Correlation Network

**Figure 4:**
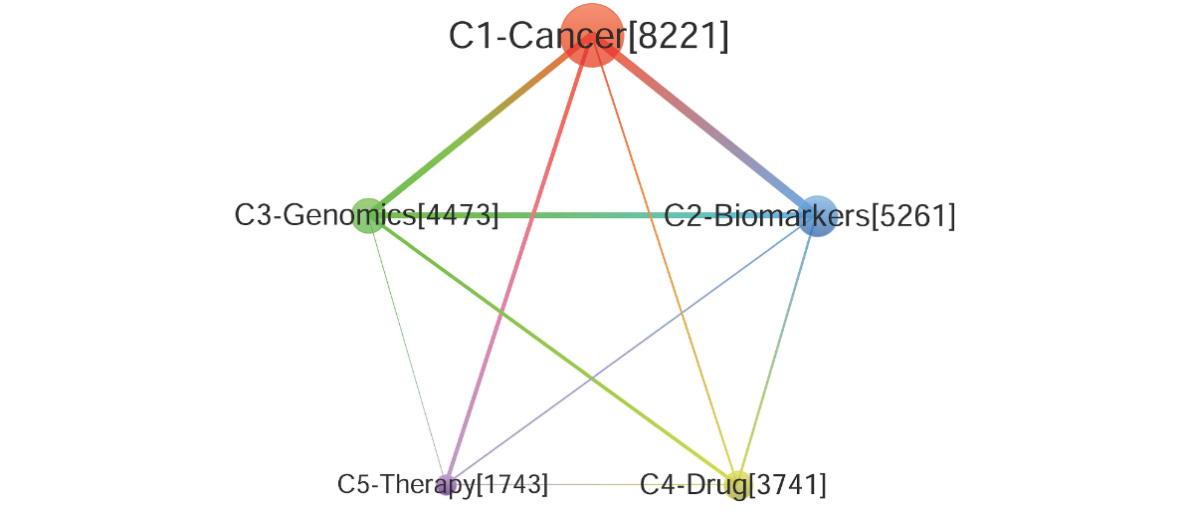
Correlation structure of theme communities in PM research.

The structural characteristics of PM research need to be further assessed by the visualization of its coword networks. As shown in Figure 4, each node represents one theme community or research subdirection. The size of the node, determined by the sum of the frequency of all words in the community, represents the scale of this direction. Each edge represents the correlation between the theme communities. Thicker edges indicate greater correlation strengths and a greater influence between communities. In general, C1-Cancer, C2-Biomarkers, C3-Genomics, and C4-Drug have formed a closely related and stable research structure; however, C5-Therapy, loosely correlated with the communities mentioned above, is considered an isolated and marginal research direction. It is noteworthy that the C1-Cancer community has the highest correlation with other communities, highlighting its important position and influence in the entire PM research field. Particularly, C1 has shown that its correlation strength with C2 and C3 is at the highest level. The 3 communities above can be regarded as core directions of PM research, which have the strongest interaction with and influence on each other. In addition, the correlation between the C1 and C5 communities is also strong, indicating interactions between the 2 research directions of Cancer and Therapy, as well as Genomics and Drug.

**Figure 5:**
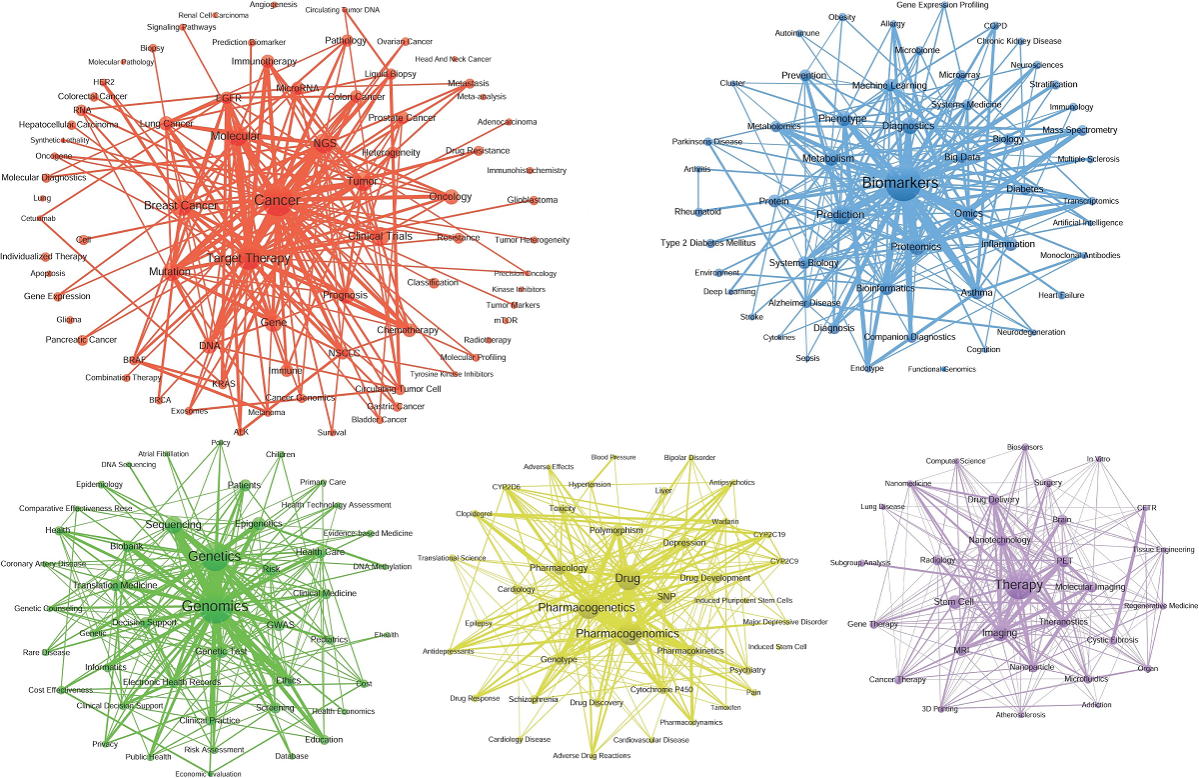
A total of 5 theme communities in precision medicine research. EGFR: epidermal growth factor receptor; NGS: next-generation sequencing; NSCLC: non–small cell lung cancer.

Furthermore, in terms of the internal correlation of the research themes community (Table 6 and Figure 5), especially regarding the indices of the average degree and density, the degree centrality of C1-Cancer and C2-Biomarkers is the highest. Second, C3-Genomics and C4-Drug are subcore research themes, whereas C5-Therapy is a self-contained research theme in PM research but in a marginal position. Finally, C1-Cancer theme community is the most closely correlated within the community and the most mature subject direction in this PM research. The other thematic communities are also closely correlated within them and have a relatively mature development. Overall, the density of all PM research topic communities is higher than the overall density of the coword network. Each research direction has been self-contained and well-developed. However, the strength of the correlation between communities is much weaker than that within the community. The results show that PM research directions are significantly differentiated and that the correlations and interactions between communities are generally insufficient.

**Table 6:** Indicators of 5 theme communities in precision medicine research.

Community	Number of nodes	Number of edges	Total frequency	Average degree	Density
C1-Cancer	76	1535	8221	82.8026	0.5386
C2-Biomarkers	53	652	5261	78.6792	0.4731
C3-Genomics	45	469	4473	70.7778	0.4737
C4-Drug	40	385	3741	68.375	0.4936
C5-Therapy	30	211	2743	65.7667	0.4851

### Evolution of and Trends in Precision Medicine Research

The bibliographic data were divided with the year as the unit of time, and an evolution graph was generated to reveal the evolutionary patterns of PM research. In addition, based on centrality and density, theme communities were graphed in a strategic graph (a 2D map). The relative status and development trend of each theme community in the PM research were revealed.

#### Evolution Venation of Precision Medicine Research

##### Overview

**Figure 6:**
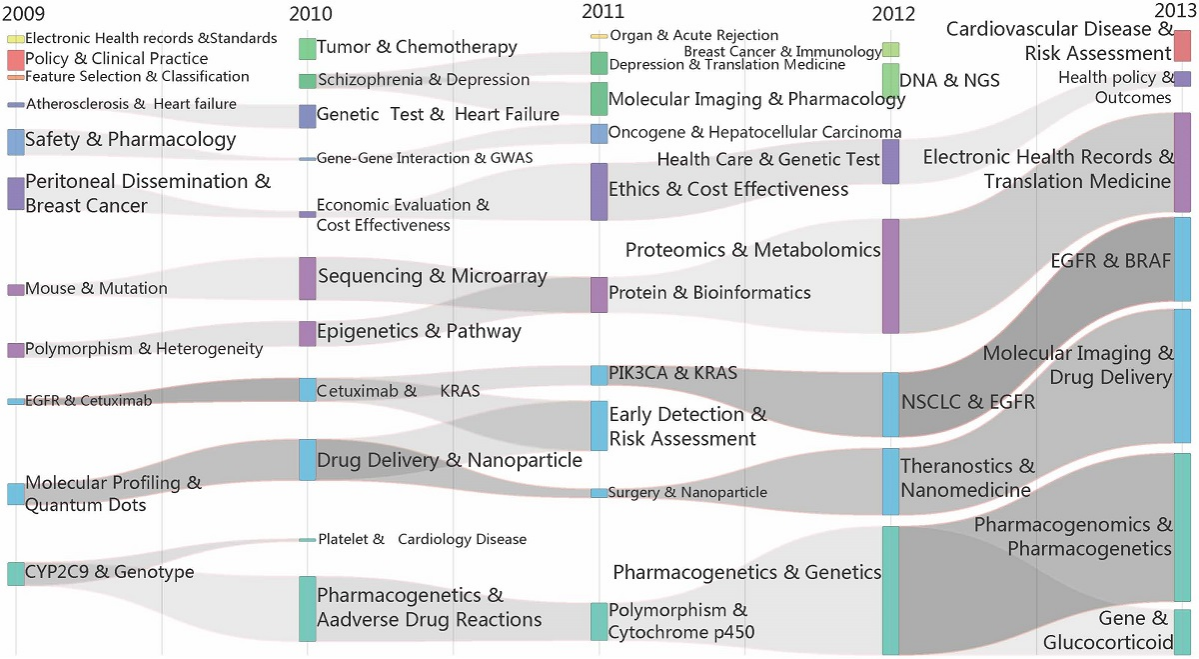
The evolution of theme communities of precision medicine research over time (2009-2013). ALK: ALK Receptor Tyrosine Kinase; BRAF: v-raf murine sarcoma viral oncogene homolog B1; BRCA: BReast CAncer gene; EGFR: Epidermal growth factor receptor; HER2: Receptor tyrosine-protein kinase erbB-2; NGS: Next-generation sequencing; KRAS: Ki-ras2 Kirsten rat sarcoma viral oncogene homolog; mTOR: The mammalian target of rapamycin; NSCLC: Non–small cell lung cancer.

**Figure 7:**
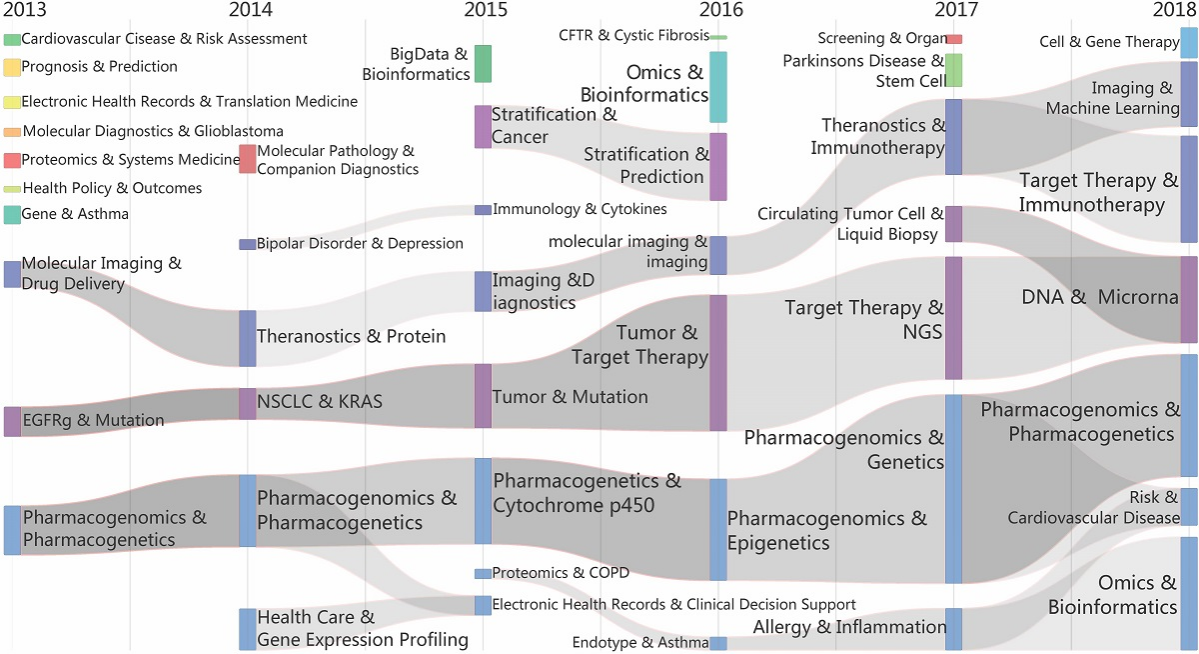
The evolution of theme communities of precision medicine research over time (2013-2018). ALK: ALK Receptor Tyrosine Kinase; BRAF: v-raf murine sarcoma viral oncogene homolog B1; CYP2C9: Cytochrome P450 2C9; EGFR: Epidermal growth factor receptor; GWAS: genome-wide association study; KRAS: Ki-ras2 Kirsten rat sarcoma viral oncogene homolog; NGS: Next-generation sequencing; NSCLC: Non–small cell lung cancer; PIK3CA: phosphatidylinositol-4,5-bisphosphate 3-kinase, catalytic subunit alpha.

To clearly show the development, the evolution of PM research was divided into 2 stages, namely, Stage 1 (2009-2013) and Stage 2 (2014-2018), as shown in Figures 6 and 7. Tubes are colored in each year to represent different topic communities. They are linked because of overlap in keywords in 2 adjacent years, and the evolution venations will be generated with the same color as shown in the figures. According to variations in the topics, such as overlapping, differentiation and fusion of topics, and isolation, we aimed to determine the developing trends of PM. In the past ten years, the continuity in PM themes has generally been good, and a consensus on research directions has formed. Moreover, research in PM has deepened and expanded, especially in Stage 2 (2014-2018), where PM research has maintained good continuity. In the same period, there was more differentiation and integration of the research areas; the interaction between the subjects of the studies was also more pronounced.

##### Stage 1 (2009-2013)

First, there are 4 obvious thematic evolutions: the Pharmacogenomics and Pharmacogenetics venation (including Pharmacogenomics, Genetics, Polymorphism, Adverse Drug Reactions, and CYP2C9), the epidermal growth factor receptor (EGFR) and v-raf murine sarcoma viral oncogene homolog B1 (BRAF) venation (including Molecular Imaging, Drug Delivery, non–small-cell lung cancer [NSCLC], and Ki-ras2 Kirsten rat sarcoma viral oncogene homolog [KRAS]), the Proteomics and Metabolomics venation (including Sequencing, Bioinformatics, and Translation Medicine), and the Ethics and Cost-Effectiveness venation (including Health Care, Genetic Test, Health Policy, and Breast Cancer).

Each venation is independent and less differentiated, and the internal system for the theme communities is relatively mature. The Pharmacogenomics and Pharmacogenetics venation and the EGFR and BRAF venation are larger scale, so they can thus be considered the 2 important research directions in this period. The evolution of some themes, such as Schizophrenia and Oncogenes, has been interrupted, which may be due to the lack of continuous concern about such subjects or their integration into other subjects. We also find that there are a few isolated themes during different periods, such as Policy, Clinical Practice, Tumor, Chemotherapy, Organ, and NGS. Owing to strong internal correlation, these themes have been clustered as a research direction. However, such studies have not yet formed a systematic and continuous direction.

##### Stage 2 (2014-2018)

We performed an independent analysis for the years 2013 and 2018 to discover the continuity between 2013 and 2014. There are many overlapping thematic communities in these 2 years as well as overlapping research themes, such as EGFR and BRAF, Molecular Imaging and Drugs, and Pharmacogenomics and Pharmacogenetics, which exhibit good continuity. Overall, the sustainability and stability of PM research in this stage are better than that in Stage 1. Research on PM in terms of themes is more concentrated, which indicates the more consistent and mature direction of progression.

According to the evolutionary graph, there are 3 major research themes at this stage: Molecular Imaging and Drug Delivery, EGFR and Mutation, and Pharmacogenomics and Pharmacogenetics. First, the Molecular Imaging and Drug Delivery venation includes Theranostics, Diagnostics, Immunotherapy, and Machine Learning. The EGFR and Mutation venation includes NSCLC, KRAS, Tumor, Target Therapy, NGS, DNA, and MicroRNA. The Pharmacogenomics and Pharmacogenetics venation includes Cytochrome P450, Epigenetics, Cardiovascular Disease, Omics, and Bioinformatics. Simultaneously, Stratification and Prediction and related topics have also formed an independent evolutionary venation. Although small in scale, they have also become a self-contained system. However, there are also discontinuous evolutions and isolated topics at this stage, such as the evolution of Bipolar Disorder, which was interrupted in 2015. In this period, Parkinson Disease, Stem Cell, and Big Data finally become isolated research themes rather than evolutionary venations.

#### Development Trends in Precision Medicine Research

**Figure 8:**
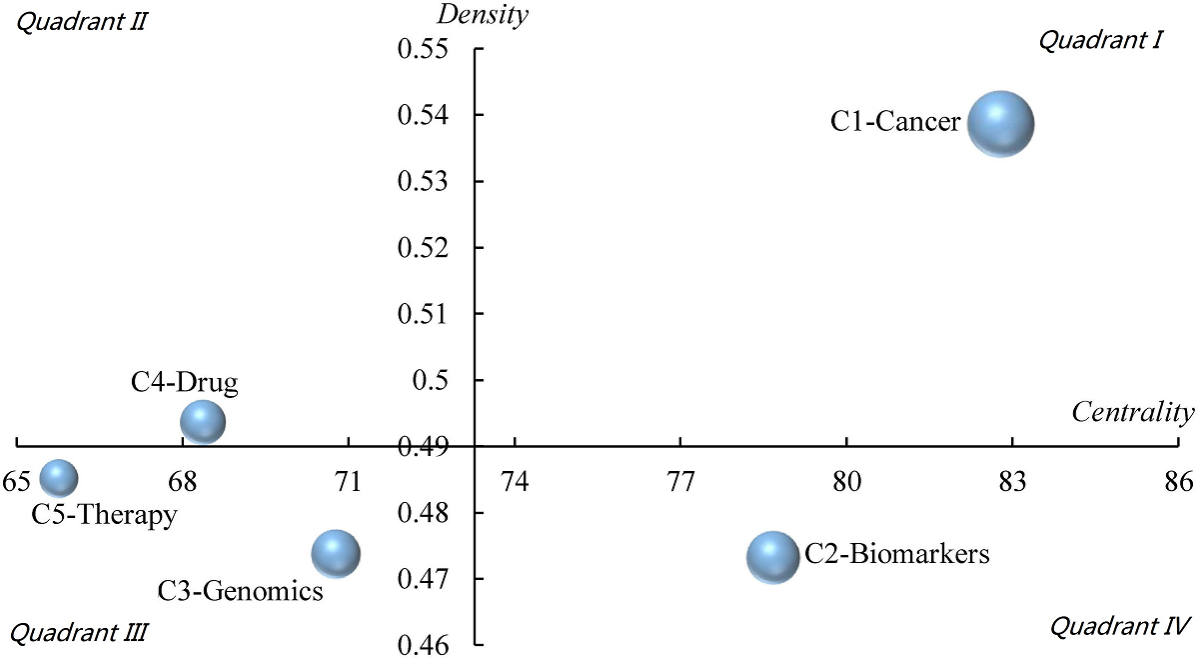
The relative development status and trends of 5 theme communities in the strategic diagram.

The theme community in the PM study is distributed in the strategic map according to centrality and density (Figure 8). On the basis of indicator analysis of the theme communities, we found that C1 is in the first quadrant. Its centrality degree and density are relatively high, indicating that it is the core and mature direction of PM research. C4 is in the second quadrant, with low centrality degree and high density. It can be considered to be the mature direction, but not the core of PM research. In the third quadrant, C3 and C5, with both low centrality and density, are not the core directions and are not mature. However, C3 is close to the origin of density, which means it has the potential to be the core of PM research and that it will develop into a mature community. C2 is in the fourth quadrant, the centrality of which is high, but the density is low. C2 can be considered the core direction, but it is generally immature or involves too many topics.

## Discussion

### Principal Findings

Based on the results, it is possible for us to better understand the main research directions of PM research and accurately evaluate its importance, maturity, and interactions. First, we determined that overall work in PM research is unbalanced but that the theme community is balanced. As PM was newly born as an independent academic subject, researchers paid most of their attention to only a few popular words, such as Biomarkers, Genomics, Cancer, Therapy, Genetics, Drug, Target Therapy, Pharmacogenomics, Pharmacogenetics, and Molecular. The words mentioned above can be classified into the following categories: The applied subject (Cancer), The associated technology and research (Biomarkers, Genomics, and Genetics), pharmacology (Pharmacogenomics), and clinical practice (Treatment, Risk Prediction, Molecular Target Treatment, and Diagnosis). These words not only reflect areas of scientific concern, but more importantly, they indicate the major research directions of PM. However, we also found that the attention paid to most research themes is relatively dispersed. We could speculate that the current status of PM research is possibly as follows: (1) the most mature application of PM is in the subject of Oncology; (2) scientists are interested in discovering Biomarkers, mainly using genomics and genetic methods; (3) pharmacology is an important interdisciplinary field involved with PM, with the aim to make drug utility safer and more efficient; and (4) PM is widely used in Clinical Medicine, including for consulting, diagnosis, and treatment (especially molecular target treatment).

With the visualization of the coword network, we found that the themes were more inclined to be clustered around other popular minority keywords. Thus, the theme communities, both well-layered and balanced-scaled, were finally formed. The communities included C1-Cancer, C2-Biomarkers, C3-Genomics, C4-Drug, and C5-Therapy. According to the analysis of correlation between the theme’s communities, we can draw the following inferences: C1-Cancer, as the largest community, indicates that the application of PM in Clinical Oncology might already be mature. The other directions, such as technical studies and Clinical Medicine, are widely associated with Cancer. C2-Biomarkers is the second largest group and plays a key role as the basis of PM research. Scientists still strive for biomarker discovery with various techniques and for the transformation of these discoveries into clinical therapeutics and the prediction of clinical outcomes [61,62]. Owing to significant progress in Genomics and Pharmacology, C4-Drug community, as an independent community, indicates special concern by both pharmacologists and clinicians. In this area, scientists are trying to explore the genetic correlation of Pharmacology and Genomics. These findings will be the foundation of PM, improving drug efficacy and safety [63,64]. It is also noteworthy that the development time of C4-Drug is short, but the fastest. Significant progress has been made in Genomics, Pharmacological Dynamics, Pharmacology, and Metabolomics, and these disciplines are playing an increasingly important role in the field. Although C3-Genomics is relatively isolated and not at the core of PM research, Genomics is one of the most important methods of detecting Biomarkers and is still widely used in various fields of PM [65]. Its decline is due to the application of new technologies, such as high-throughput Omics [66] and Molecular Imaging technology [67]. C5-Therapy, independent but of the smallest scale, indicates that individualized treatment is the ultimate goal of PM, resulting in this aspect gaining the attention of scientists. However, the strategy for treatment is still far from well-developed, which proves the limited scale of the community. According to the major themes included in the C5-Therapy community, individualized treatment mainly involves traditional strategies such as Surgery, Chemotherapy, and Radiology. Interestingly, new methods such as gene therapy, stem cell, and tissue engineering have been available in PM treatment. On the other hand, PET and Molecular Imaging are new technologies that can be applied for stratifications. Through the strategic diagram, C5-Therapy is noncore in PM research; however, we can infer that while the community has not yet matured, it is of great potential.

Through the analysis of the evolution of theme communities over time, PM research has a clear evolutionary and developmental trend. In 2 stages of evolution, we have discovered a large number of well-concentrated evolutionary pathways, which indicates the maturity of PM. The theme community in PM research is well-structured and contains the core and promising directions, such as Biomarkers, Pharmacogenomics, MicroRNA, Imaging, and even Machine Learning. We also identified a dramatic development in techniques and pharmacology directions. It is worth noting that the trend toward PM in nononcology diseases has the potential to become mature, and NSCLC could develop to become an independent and mature venation. It indicates that the application of PM in NSCLC is relatively mature. Clinicians have applied strategies or technologies involved with PM, such as Biomarker, Molecular Imaging, and Pharmacogenomics, to achieve precise treatment [68-70].

### Limitations

Our study reveals the structure and developmental trends of PM research from the perspective of keywords and their relationships. To some extent, this study provides insight into PM research; however, there are still limitations to this work. Regarding the research sample, this study used the literature to reveal the development status of PM. This research method could be regarded as a reasonable and cost-effective strategy rather than a comprehensive and accurate way to evaluate the true status of PM research.

### Conclusions and Future Directions

Our study reveals the hotspots, structures, evolutions, and developmental trends of PM research in the past 10 years by means of social network analysis and visualization. We also made the following valuable discoveries: (1) using a graph, the network can describe, in detail, the development of PM research; and (2) the network uncovers the relationship between the themes and the intrinsic mechanism about how they interact, which could provide insights into future research directions.

In the future, we will perform data mining on the content of PM-related literature (eg, reports and illness records) to better reveal the condition of the entire network from various perspectives. In terms of research methods, based on previous work, the efficacy of coword analysis has been identified. Our study also validates this research method, and using it, we were able to obtain some valuable discoveries. In future studies, we aim to perform a further, comprehensive assessment of PM research through various perspectives, such as interdisciplinary research and institutes.

## Abbreviations

2D: two-dimensional
BRAF: v-raf murine sarcoma viral oncogene homolog B1
EGFR: epidermal growth factor receptor
HER2: human epidermal growth factor receptor 2
KRAS: Ki-ras2 Kirsten rat sarcoma viral oncogene homolog
NGS: next-generation sequencing
NSCLC: non–small cell lung cancer
P4 medicine: predictive, preventative, personalized, and participatory medicine
PET: positron emission tomography
PM: precision medicine
WOSCC: Web of Science Core Collection

## References

[1] Mohler J, Najafi B, Fain M, Ramos KS (2015). Precision medicine: a wider definition. J Am Geriatr Soc.

[2] Geyer FC, Lopez-Garcia MA, Lambros MB, Reis-Filho JS (2009). Genetic characterization of breast cancer and implications for clinical management. J Cell Mol Med.

[3] Luttropp K, Lindholm B, Carrero JJ, Glorieux G, Schepers E, Vanholder R, Schalling M, Stenvinkel P, Nordfors L (2009). Genetics/Genomics in chronic kidney disease--towards personalized medicine?. Semin Dial.

[4] Aronson SJ, Rehm HL (2015). Building the foundation for genomics in precision medicine. Nature.

[5] Kohler I, Hankemeier T, van der Graaf PH, Knibbe CA, van Hasselt JG (2017). Integrating clinical metabolomics-based biomarker discovery and clinical pharmacology to enable precision medicine. Eur J Pharm Sci.

[6] Kuntz TM, Gilbert JA (2017). Introducing the microbiome into precision medicine. Trends Pharmacol Sci.

[7] Duarte TT, Spencer CT (2016). Personalized proteomics: the future of precision medicine. Proteomes.

[8] Schwaederle M, Zhao M, Lee JJ, Eggermont AM, Schilsky RL, Mendelsohn J, Lazar V, Kurzrock R (2015). Impact of precision medicine in diverse cancers: a meta-analysis of phase II clinical trials. J Clin Oncol.

[9] Printz C (2009). National Institutes of Health releases new guidelines for stem cell research. Cancer.

[10] Hollingsworth SJ (2015). Precision medicine in oncology drug development: a pharma perspective. Drug Discov Today.

[11] He M, Xia J, Shehab M, Wang X (2015). The development of precision medicine in clinical practice. Clin Transl Med.

[12] He Q (1999). Knowledge discovery through co-word analysis. Libr Trends.

[13] Li F, Li M, Guan P, Ma S, Cui L (2015). Mapping publication trends and identifying hot spots of research on Internet health information seeking behavior: a quantitative and co-word biclustering analysis. J Med Internet Res.

[14] Chang X, Zhou X, Luo L, Yang C, Pan H, Zhang S (2017). Hotspots in research on the measurement of medical students' clinical competence from 2012-2016 based on co-word analysis. BMC Med Educ.

[15] Leung XY, Sun J, Bai B (2017). Bibliometrics of social media research: a co-citation and co-word analysis. Int J Hosp Manag.

[16] Li X, Qiao H, Wang S (2017). Exploring evolution and emerging trends in business model study: a co-citation analysis. Scientometrics.

[17] Chaker AM, Klimek L (2015). [Individualized, personalized and stratified medicine: a challenge for allergology in ENT?]. HNO.

[18] Sobradillo P, Pozo F, Agustí A (2011). P4 Medicine: the Future Around the Corner. Arch Bronconeumol.

[19] Manolio TA, Green ED (2014). Leading the way to genomic medicine. Am J Med Genet C Semin Med Genet.

[20] Ashley EA (2015). The precision medicine initiative: a new national effort. J Am Med Assoc.

[21] Ogino S, Nishihara R, VanderWeele TJ, Wang M, Nishi A, Lochhead P, Qian ZR, Zhang X, Wu K, Nan H, Yoshida K, Milner DA, Chan AT, Field AE, Camargo CA, Williams MA, Giovannucci EL (2016). Review article: the role of molecular pathological epidemiology in the study of neoplastic and non-neoplastic diseases in the era of precision medicine. Epidemiology.

[22] Slamon DJ, Leyland-Jones B, Shak S, Fuchs H, Paton V, Bajamonde A, Fleming T, Eiermann W, Wolter J, Pegram M, Baselga J, Norton L (2001). Use of chemotherapy plus a monoclonal antibody against HER2 for metastatic breast cancer that overexpresses HER2. N Engl J Med.

[23] Antoniou A, Pharoah PD, Narod S, Risch HA, Eyfjord JE, Hopper JL, Loman N, Olsson H, Johannsson O, Borg A, Pasini B, Radice P, Manoukian S, Eccles DM, Tang N, Olah E, Anton-Culver H, Warner E, Lubinski J, Gronwald J, Gorski B, Tulinius H, Thorlacius S, Eerola H, Nevanlinna H, Syrjäkoski K, Kallioniemi O, Thompson D, Evans C, Peto J, Lalloo F, Evans DG, Easton DF (2003). Average risks of breast and ovarian cancer associated with *BRCA1* or *BRCA2* mutations detected in case Series unselected for family history: a combined analysis of 22 studies. Am J Hum Genet.

[24] Mancinelli L, Cronin M, Sadée W (2000). Pharmacogenomics: the promise of personalized medicine. AAPS PharmSci.

[25] Shord SS (2017). The role of clinical pharmacology in oncology dose selection: advances and opportunities in personalized medicine. J Clin Pharmacol.

[26] Roper N, Stensland KD, Hendricks R, Galsky MD (2015). The landscape of precision cancer medicine clinical trials in the United States. Cancer Treat Rev.

[27] Fiore RN, Goodman KW (2016). Precision medicine ethics: selected issues and developments in next-generation sequencing, clinical oncology, and ethics. Curr Opin Oncol.

[28] Trosman JR, Weldon CB, Douglas MP, Kurian AW, Kelley RK, Deverka PA, Phillips KA (2017). Payer coverage for hereditary cancer panels: barriers, opportunities, and implications for the precision medicine initiative. J Natl Compr Canc Netw.

[29] McPadden J, Durant TJ, Bunch DR, Coppi A, Price N, Rodgerson K, Torre CJ, Byron W, Hsiao AL, Krumholz HM, Schulz WL (2019). Health care and precision medicine research: analysis of a scalable data science platform. J Med Internet Res.

[30] Krittanawong C, Zhang H, Wang Z, Aydar M, Kitai T (2017). Artificial intelligence in precision cardiovascular medicine. J Am Coll Cardiol.

[31] Hu J, Zhang Y (2017). Discovering the interdisciplinary nature of Big Data research through social network analysis and visualization. Scientometrics.

[32] Chen R, Snyder M (2013). Promise of personalized omics to precision medicine. Wiley Interdiscip Rev Syst Biol Med.

[33] Lu Z, Minko T (2017). Molecular imaging for precision medicine. Adv Drug Deliv Rev.

[34] Davis T (2012). Biomedical, Bio-Nano, Personalized Medicine – It's All Nanomedicine to Us!. Aust J Chem.

[35] Vieta E (2015). [Personalised medicine applied to mental health: precision psychiatry]. Rev Psiquiatr Salud Ment.

[36] Krittanawong C (2017). Future physicians in the era of precision cardiovascular medicine. Circulation.

[37] Oberle AJ, Mathur P (2017). Precision medicine in asthma: the role of bronchial thermoplasty. Curr Opin Pulm Med.

[38] Fischer S, Neurath MF (2017). Precision medicine in inflammatory bowel diseases. Clin Pharmacol Ther.

[39] Korngiebel DM, Thummel KE, Burke W (2017). Implementing precision medicine: the ethical challenges. Trends Pharmacol Sci.

[40] Brothers KB, Rothstein MA (2015). Ethical, legal and social implications of incorporating personalized medicine into healthcare. Per Med.

[41] Duffy DJ (2016). Problems, challenges and promises: perspectives on precision medicine. Brief Bioinform.

[42] Loncar-Turukalo T, Zdravevski E, Machado da Silva J, Chouvarda I, Trajkovik V (2019). Literature on wearable technology for connected health: scoping review of research trends, advances, and barriers. J Med Internet Res.

[43] Wei W, Shi B, Guan X, Ma J, Wang Y, Liu J (2019). Mapping theme trends and knowledge structures for human neural stem cells: a quantitative and co-word biclustering analysis for the 2013-2018 period. Neural Regen Res.

[44] Hu J, Zhang Y (2015). Research patterns and trends of Recommendation System in China using co-word analysis. Inf Process Manage.

[45] Hu C, Hu J, Deng S, Liu Y (2013). A co-word analysis of library and information science in China. Scientometrics.

[46] Börner K (2011). Plug-and-play macroscopes. Commun ACM.

[47] Hu J, Zhang Y (2017). Structure and patterns of cross-national Big Data research collaborations. J Doc.

[48] Leydesdorff L, de Moya-Anegón F, Guerrero-Bote VP (2015). Journal maps, interactive overlays, and the measurement of interdisciplinarity on the basis of Scopus data (1996-2012). J Assn Inf Sci Tech.

[49] Hu J, Huang R, Wang Y (2018). Geographical visualization of research collaborations of library science in China. Electron Libr.

[50] Doreian P, Lloyd P, Mrvar A (2013). Partitioning large signed two-mode networks: problems and prospects. Soc Netw.

[51] Callon M, Courtial JP, Laville F (1991). Co-word analysis as a tool for describing the network of interactions between basic and technological research: The case of polymer chemsitry. Scientometrics.

[52] Albert R, Barabási AL (2002). Statistical mechanics of complex networks. Rev Mod Phys.

[53] Chen G, Xiao L (2016). Selecting publication keywords for domain analysis in bibliometrics: a comparison of three methods. J Inform.

[54] Blondel VD, Guillaume J, Lambiotte R, Lefebvre E (2008). Fast unfolding of communities in large networks. J Stat Mech.

[55] Muñoz-Leiva F, Viedma-del-Jesús MI, Sánchez-Fernández J, López-Herrera AG (2012). An application of co-word analysis and bibliometric maps for detecting the most highlighting themes in the consumer behaviour research from a longitudinal perspective. Qual Quant.

[56] Chen Y, Fang S (2014). Mapping the evolving patterns of patent assignees’ collaboration networks and identifying the collaboration potential. Scientometrics.

[57] van Eck NJ, Waltman L (2010). Software survey: VOSviewer, a computer program for bibliometric mapping. Scientometrics.

[58] Rosvall M, Bergstrom CT (2010). Mapping change in large networks. PLoS One.

[59] Leydesdorff L, Goldstone RL (2014). Interdisciplinarity at the journal and specialty level: The changing knowledge bases of the journal. J Assoc Inf Sci Tech.

[60] Leydesdorff L, Park HW, Wagner C (2014). International coauthorship relations in the Social Sciences Citation Index: Is internationalization leading the Network?. J Assoc Inf Sci Tech.

[61] Wang E, Cho WC, Wong SC, Liu S (2017). Disease biomarkers for precision medicine: challenges and future opportunities. Genomics Proteomics Bioinformatics.

[62] Collins DC, Sundar R, Lim JS, Yap TA (2017). Towards precision medicine in the clinic: from biomarker discovery to novel therapeutics. Trends Pharmacol Sci.

[63] Lauschke VM, Milani L, Ingelman-Sundberg M (2017). Pharmacogenomic biomarkers for improved drug therapy-recent progress and future developments. AAPS J.

[64] Relling MV, Evans WE (2015). Pharmacogenomics in the clinic. Nature.

[65] Carrasco-Ramiro F, Peiró-Pastor R, Aguado B (2017). Human genomics projects and precision medicine. Gene Ther.

[66] Kim D, Kim Y, Son N, Kang C, Kim A (2017). Recent omics technologies and their emerging applications for personalised medicine. IET Syst Biol.

[67] Wright CL, Binzel K, Zhang J, Knopp MV (2017). Advanced functional tumor imaging and precision nuclear medicine enabled by digital pet technologies. Contrast Media Mol Imaging.

[68] Hofman P (2017). ALK in non-small cell lung cancer (NSCLC) pathobiology, epidemiology, detection from tumor tissue and algorithm diagnosis in a daily practice. Cancers (Basel).

[69] Bahce I, Yaqub M, Smit EF, Lammertsma AA, van Dongen GA, Hendrikse NH (2017). Personalizing NSCLC therapy by characterizing tumors using TKI-PET and immuno-PET. Lung Cancer.

[70] Yin J, Li X, Zhou H, Liu Z (2016). Pharmacogenomics of platinum-based chemotherapy sensitivity in NSCLC: toward precision medicine. Pharmacogenomics.

